# *In Vivo* Activation of the Intracrine Vitamin D Pathway in Innate Immune Cells and Mammary Tissue during a Bacterial Infection

**DOI:** 10.1371/journal.pone.0015469

**Published:** 2010-11-29

**Authors:** Corwin D. Nelson, Timothy A. Reinhardt, Donald C. Beitz, John D. Lippolis

**Affiliations:** 1 Diseases and Immunology Research Unit, National Animal Disease Center, Agriculture Research Service, United States Department of Agriculture, Ames, Iowa, United States of America; 2 Department of Biochemistry, Biophysics and Molecular Biology, Iowa State University, Ames, Iowa, United States of America; 3 Department of Animal Science, Iowa State University, Ames, Iowa, United States of America; New York University, United States of America

## Abstract

Numerous *in vitro* studies have shown that toll-like receptor signaling induces 25-hydroxyvitamin D_3_ 1α-hydroxylase (1α-OHase; CYP27B1) expression in macrophages from various species. 1α-OHase is the primary enzyme that converts 25-hydroxyvitamin D_3_ to 1,25-dihydroxyvitamin D_3_ (1,25(OH)_2_D_3_). Subsequently, synthesis of 1,25(OH)_2_D_3_ by 1α-OHase in macrophages has been shown to modulate innate immune responses of macrophages. Despite the numerous *in vitro* studies that have shown 1α-OHase expression is induced in macrophages, however, evidence that 1α-OHase expression is induced by pathogens *in vivo* is limited. The objective of this study was to evaluate 1α-OHase gene expression in macrophages and mammary tissue during an *in vivo* bacterial infection with *Streptococcus uberis*. In tissue and secreted cells from the infected mammary glands, 1α-OHase gene expression was significantly increased compared to expression in tissue and cells from the healthy mammary tissue. Separation of the cells by FACS9 revealed that 1α-OHase was predominantly expressed in the CD14^+^ cells isolated from the infected mammary tissue. The 24-hydroxylase gene, a gene that is highly upregulated by 1,25(OH)_2_D_3_, was significantly more expressed in tissue and cells from the infected mammary tissue than from the healthy uninfected mammary tissue thus indicating significant local 1,25(OH)_2_D_3_ production at the infection site. In conclusion, this study provides the first *in vivo* evidence that 1α-OHase expression is upregulated in macrophages in response to bacterial infection and that 1α-OHase at the site of infection provides 1,25(OH)_2_D_3_ for local regulation of vitamin D responsive genes.

## Introduction

Vitamin D has been shown to have a role in regulating immune function in addition to the well-known role it has in regulating calcium homeostasis. 1,25-dihydroxyvitamin D_3_ (1,25(OH)_2_D_3_), the active vitamin D_3_ metabolite, regulates the expression of several genes involved in host defense and immune function [1]. Therefore, synthesis of 1,25(OH)_2_D_3_ to control vitamin D responsive genes in immune cells is a critical factor in regulating of immune function.

The enzyme that synthesizes 1,25(OH)_2_D_3_ from 25-hydroxyvitamin D_3_ (25(OH)D_3_) is 1α-hydroxylase (1α-OHase; CYP27B1) [2]. In the kidney, 1α-OHase expression is induced by parathyroid hormone in response to calcium homeostasis [3], [4]. Synthesis of 1,25(OH)_2_D_3_ in the kidney regulates the circulating concentration of 1,25(OH)_2_D_3_ and the endocrine actions of vitamin D. In monocytes and macrophages, 1α-OHase is expressed in response to activation by IFN-γ or TLR signaling [5], [6], [7], [8]. Conversion of 25(OH)D_3_ to 1,25(OH)_2_D_3_ by 1α-OHase in monocytes regulates the expression of vitamin D responsive genes in an intracrine manner [9]. In human monocytes, production of 1,25(OH)_2_D_3_ by 1α-OHase drives cathelicidin gene expression [6]. In the same way, 1α-OHase activity in bovine monocytes enhances iNOS and RANTES gene expression [8]. From in vitro studies, expression of 1α-OHase by macrophages at the site of an infection seems to be an important part of innate immunity. Montoya et al have shown that upregulation of the vitamin D pathway occurs in leprosy lesions of patients with self limiting forms of the disease [10], however, beyond that study there is no evidence that 1α-OHase is expressed by macrophages in vivo as a result of experimental infection [11].

Intra-mammary infections during lactation offers a model of bacterial infection to determine if 1α-OHase is expressed in response to bacterial infection in vivo. Common pathogens that cause mammary infections include *Escherichia coli*, *Staphylococcus aureus*, and *Streptococcus uberis* [12], [13], [14], [15]. During mammary infection the number of somatic cells secreted in milk will often exceed 10^6^ cells/mL. Approximately 80 to 90 percent of somatic cells in milk from an infected mammary gland are neutrophils and the remainder of the cells are macrophages and lymphocytes [16]. The advantage of using a mammary infection model is that the infiltrating cells during mammary infection can easily isolated from milk using non-invasive procedures; allowing us to study the in vivo immune responses of immune cells to bacterial infection.

TLRs are present in the bovine mammary gland [17] and invasion of the mammary gland by bacteria triggers an innate immune response by TLR signaling [18]. Based on in vitro evidence that 1α-OHase expression in macrophages is induced by TLR recognition of bacteria [6], [8], [19], we hypothesized that 1α-OHase expression would be upregulated in both macrophages and mammary tissue during a mammary infection. Using an intra-mammary infection as a model in vivo bacterial infection, we present the first in vivo evidence that 1α-OHase expression is upregulated in CD14^+^ cells that are at the site of a bacterial infection. The subsequent large increased expression of 24-hydroxylase (24-OHase) at the infection site supports local in vivo production of 1,25(OH)_2_D_3_ at the site of an infection.

## Materials and Methods

### Animals

Eight, mid-lactation Holstein cows at the USDA National Animal Disease Center were used for this study. The National Animal Disease Center animal care and use committee approved all procedures used in this study (Protocol ARS-4001). Prior to the study, all cows were healthy and bacteria were not detected in their milk. Mammary infection was induced by infusion of 500 cfu of *Streptococcus uberis* strain 0140 (*S. uberis*; a gift from Dr. Max Paape, USDA, Beltsville, MD) suspended in 3 mL of PBS into one mammary gland. The contra lateral gland was infused with an equal volume of PBS and served as the control. The amount of *S. uberis* in the milk from the control and infected glands was determined by culturing log dilutions of milk samples on blood agar plates for 24 hours at 37°C.

### Collection of tissue and cells

Mammary tissue was collected from various locations in the control and infected glands of three cows that were euthanized at the onset of clinical mammary infection. Clinical infection was defined by rectal temperature, noticeable inflammation, and presence of bacteria in the milk. Tissue was placed in RNAlater (Qiagen, Valencia, CA), snap frozen in liquid nitrogen and stored at −80°C.

Cells were isolated from milk from the control and infected glands and peripheral blood of 5 cows before infection with *S. uberis* and at the onset of clinical mastitis. Cells were isolated from milk by centrifuging the milk at 1000Xg for 20 min. Peripheral blood leukocytes were isolated by lysing the erythrocytes with a hypotonic buffer and centrifuging at 650×g for 10 min. The cell pellets from milk and blood were washed 3X by resuspending in cold PBS and centrifuging at 650×g for 10 min. Cells were lysed with RLT buffer (Qiagen) and stored at −80°C or separated by FACS.

For separation of cells from blood and milk by FACS, cells were labeled with monoclonal anti-bovine CD14 IgG_1_ (CAM36A; VMRD, Inc., Pullman, WA) and a PE-conjugated anti-mouse IgG antibody (Southern Biotech, Birmingham, AL). Labeled cells were separated based on fluorescence intensity using the BD FACSAria Cell Sorting System (BD Biosciences, San Jose, CA). Approximately 10^6^ CD14^+^ and CD14^−^ cells with greater than 95% purity were isolated from milk from the infected gland and peripheral blood of each animal. The sorted cells were lysed with RLT buffer (Qiagen) and stored at −80°C.

### Real-time PCR

RNA was isolated from mammary tissue and cells using an RNeasy Mini Kit (Qiagen). RNA samples were eluted in 50 µL of RNAse-free water. Immediately after elution, RNA was reverse transcribed to cDNA using a High Capacity Reverse Transcription Kit (Applied Biosystems, Foster City, CA) with 10 µL RNA sample and 20 units of RNase inhibitor (RNaseOUT, Invitrogen, Carlsbad, CA) in a 20 µL reaction. Reactions were incubated at 37°C for 2 h and heated to 85°C for 5 s. The cDNA samples were diluted 1∶10 in water and stored at −20°C. Real-time PCR was performed using a 7300 Real-Time PCR System (Applied Biosystems). The reactions were incubated at 95°C for 10 min followed by 40 cycles of 95°C for 15 s and 60°C for 1 min. Each reaction contained 12.5 µL SYBR Green PCR Master Mix (Applied Biosystems), 2.5 µL each of 10 µ*M* forward and reverse primers, and 7.5 µL of diluted cDNA. Sequences for primer pairs are given in Table 1. Primers were purchased from Integrated DNA Technologies (Corralville, IA). The specificity of each primer pair was determined by gel electrophoresis of cDNA products and efficiency was determined using known dilutions of cDNA. All primer pairs, except for the VDR, have been used previously [8]. Relative gene expression was determined using the 2^-ΔΔCt^ method [20]. RPS9 was used as the reference gene. RPS9 gene expression also was compared to β-actin gene expression and the relative expression of RPS9 did not differ significantly among treatments.

**Table 1:** 

Gene (alternate name)	Accesion no.^1^	Strand	Sequence (5′ - 3′)
1α-OHase (CYP27B1)^2^	NM_001192284	ForwardReverse	TGGGACCAGATGTTTGCATTCGC TTCTCAGACTGGTTCCTCATGGCT
24-OHase (CYP24A1)^2^	NM_001191417	ForwardReverse	GAAGACTGGCAGAGGGTCAG CAGCCAAGACCTCGTTGATT
iNOS^2^	NM_001076799	ForwardReverse	GATCCAGTGGTCGAACCTGC CAGTGATGGCCGACCTGATG
RANTES (CCL5)^2^	NM_175827	ForwardReverse	CACCCACGTCCAGGAGTATT CTCGCACCCACTTCTTCTCT
RPS9^2^	NM_001101152	ForwardReverse	GTGAGGTCTGGAGGGTCAAA GGGCATTACCTTCGAACAGA
VDR	NM_001167932	ForwardReverse	AGCCACCGGCTTCCATTTCA AACAGCGCCTTCCGCTTCAT

1 Accesion numbers from NCBI database http://www.ncbi.nlm.nih.gov.

2 Primer sequences have been published previously [8].

### Statistical analysis

Statistical analysis was performed using PROC GLM of SAS (SAS Institute Inc., Cary, NC). The model used in the analysis accounted for effects of treatment and cow. ΔΔCt values were used to analyze relative gene expression. Mean ΔΔCt values ± SE were transformed (2^-ΔΔCt^) and shown as the expression relative to the control. The control treatment is designated in the figure legends. Multiple comparison tests of the means were made with the Tukey-Kramer adjustment.

## Results

### *Streptococcus uberis* mammary infection

**Figure 1:**
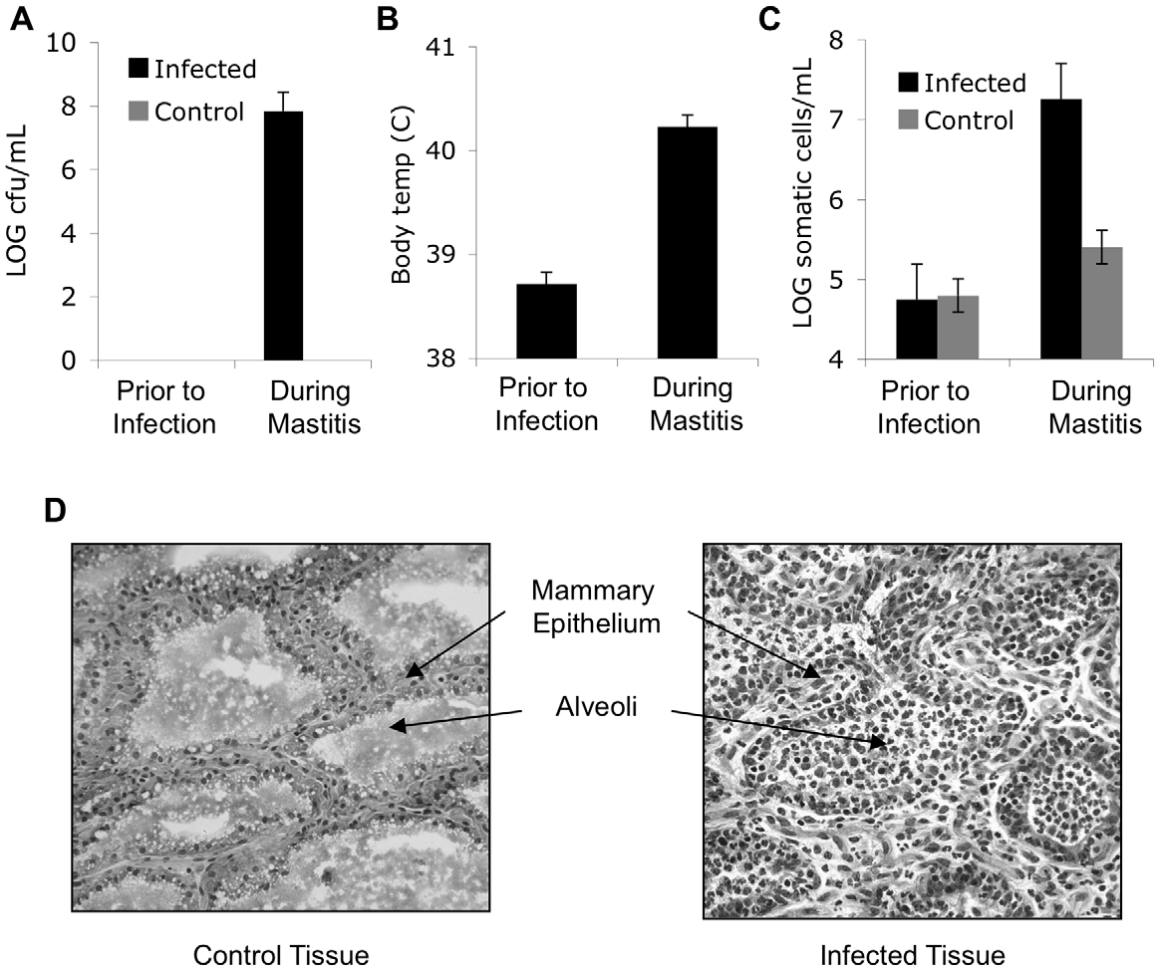
Intra-mammary infection with *Streptococcus uberis*. Five hundred CFU of *S. uberis* strain 0140 were infused into the mammary glands of 8 healthy cows. The contra-lateral glands were infused with 3 mL of PBS and served as the controls. Prior to infection, all glands were free of bacteria. The average CFU of *S. uberis* in milk (**A**), rectal temperatures (**B**), and number of somatic cells in milk (**C**) prior to and during infection are shown. Error bars represent the SE. (**D**) Representative tissue sections at 20X magnification from the control and infected mammary gland.

Eight cows were infused with 500 CFUs of *S. uberis* strain 0140 in one mammary gland and sterile PBS in the contra-lateral mammary gland. Bacteria were not detected in milk from any of the mammary glands prior to infection and were not detected in the milk from the control mammary glands during infection (Fig. 1A). The average amount of *S. uberis* in the milk from the infected mammary glands at the peak of the disease was 10^8^ CFU/mL (Fig. 1A). Prior to infection cows had normal body temperatures, but during mammary infection body temperatures were elevated (Fig. 1B). The number of somatic cells in the milk from the infected mammary glands rose from an average of 10^5^ cells/mL prior to infection to over 10^7^ cells/mL during the infection (Fig. 1C). The number of somatic cells in the control mammary glands remained near 10^5^ cells/mL on average during infection (Fig. 1C). Examination of mammary tissue by microscopy revealed inflammation and the presence of infiltrating cells in the alveoli of the infected mammary gland (Fig. 1D). The onset of clinical infection was typically 3 days after infection with *S. uberis*; which was consistent with previous studies with *S. uberis* [21]. The mean concentration of 25(OH)D in blood of the cows used in this study was 74.5 ng/ml (SEM +/−4.5) and the concentration of 25(OH)D in blood was not affected by onset of mastitis.

### 1α-Hydroxylase gene expression during mastitis

**Figure 2:**
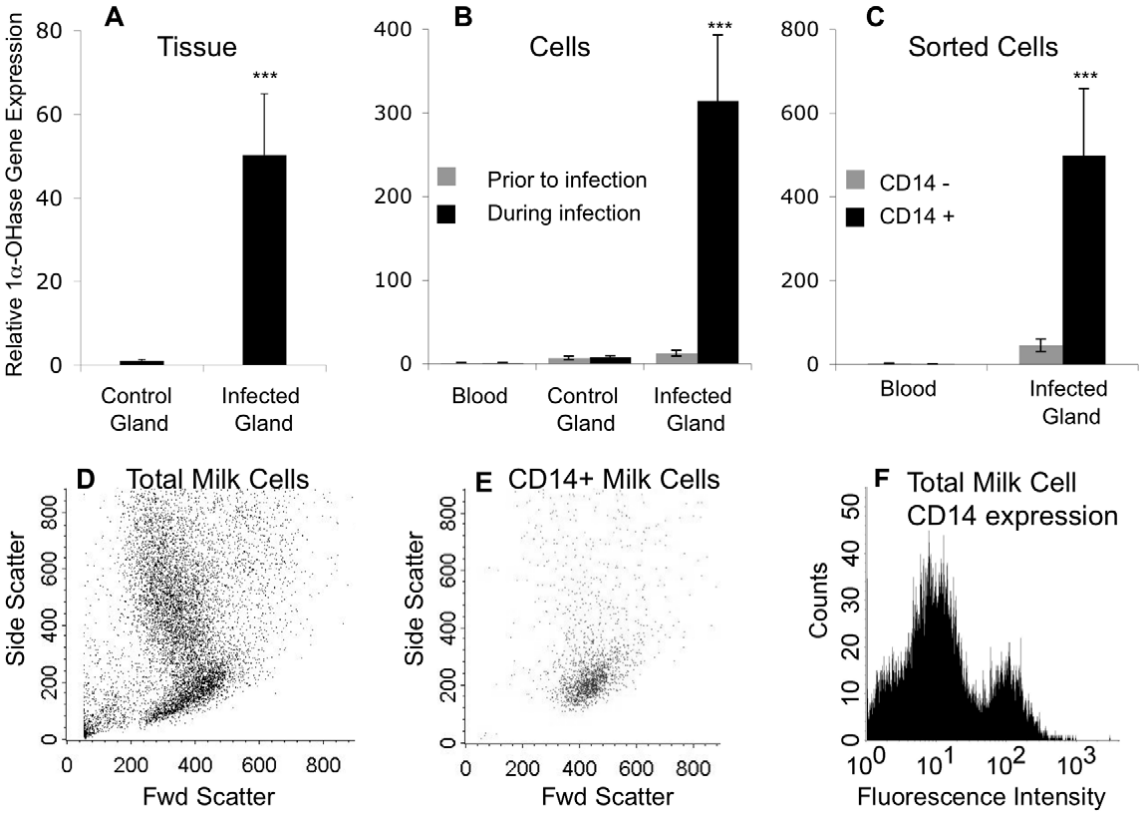
1α-OHase gene expression during *S. uberis* mammary infection. (**A–C**) Relative 1α-OHase gene expression in mammary tissue during infection (A), total cells from blood and milk prior to and during mammary infection (B), and CD14^+^ cells and CD14^−^ cells from blood and milk during mammary infection. (**A**) Three cows were infected with *S. uberis* and mammary tissue was collected from control and infected glands. (**B and C**) Five other cows were infected with *S. uberis* and cells (mononuclear and polymorphonuclear) were collected from blood and milk from the control and infected glands prior to and during infection. (**C**) Blood and milk cells from the infected gland were separated according to CD14 expression on the cell surface using FACS. The amount of 1α-OHase mRNA in each sample was determined by quantitative real-time RT-PCR and normalized to RPS9 mRNA. The relative amount of 1α-OHase mRNA was determined using the 2^-ΔΔCt^ method. Data represent the mean ± SE expression of 1α-OHase relative to 1α-OHase expression in control tissue (**A**), or peripheral blood leukocytes (**B and C**). ANOVA was performed by SAS using the general linear model and multiple comparison tests were made using the Tukey adjustment; ***mean is different from other means, *P*<0.001. (**D and E**) Scatter plots of all cells and CD14^+^ cells isolated from milk from an infected gland. (**F**) Representative histogram of CD14 expression on cells isolated from the milk from the infected gland.

In our initial experiment, secretory mammary tissue was collected from the control and infected glands of 3 cows at the onset of clinical mammary infection. In the tissue from the infected mammary glands, 1α-OHase expression was nearly 50 fold greater than 1α-OHase expression in tissue from the contra-lateral uninfected mammary glands (*P*<0.001; Fig. 2A). The alveoli in the infected mammary tissue were packed with infiltrating cells (Fig. 1D) and the infiltrating cells, which are ∼10% macrophages during acute mastitis [16], were hypothesized to be the cells expressing 1α-OHase. Therefore, to determine the contribution of infiltrating cells on 1α-OHase gene expression during mastitis we isolated cells from the milk of control and infected mammary glands from 5 cows with *S. uberis*. In cells isolated from milk from the infected mammary glands, 1α-OHase gene expression was 40 fold greater than expression in cells from the contra-lateral uninfected mammary gland (*P*<0.001) and over 300 fold greater than peripheral blood leukocytes (*P*<0.001; Fig. 2B). Finally, separating the cells according to CD14 expression revealed that 1α-OHase was predominantly expressed in the CD14^+^ cells from the infected mammary glands (*P*<0.001) compared to CD14^−^ cells from the infected gland, but not in CD14^+^ cells from peripheral blood (Fig. 2C). CD14 is a marker for monocytes and macrophages [22] and typically 10% of the cells isolated from the milk of the infected mammary gland were CD14^+^ cells (Fig. 2D–F).

### Expression of the VDR and vitamin D responsive genes during mammary infection

**Figure 3:**
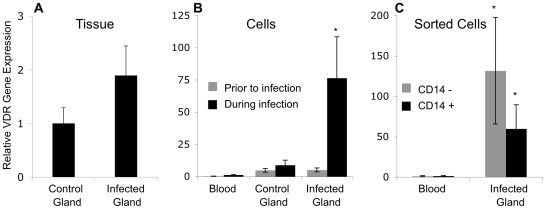
VDR gene expression during mammary *S. uberis* infection. Relative VDR gene expression in mammary tissue during an infection (**A**), total cells from blood and milk prior to and during mammary infection (**B**), and CD14^+^ cells and CD14^−^ cells from blood and milk during mammary infection (**C**). Tissue (n = 3 cows) and cells (n = 5 cows) were collected as in Figure 2. The amount of VDR mRNA in each sample was determined by quantitative real-time RT-PCR and normalized to RPS9 mRNA. The relative amount of VDR mRNA was determined using the 2^-ΔΔCt^ method. Data represent the mean ± SE expression of VDR relative to VDR expression in control tissue (**A**), or peripheral blood leukocytes (**B and C**). ANOVA was performed by SAS using the general linear model and multiple comparison tests were made using the Tukey adjustment; *mean is different from other means, *P*<0.05.

The effects of 1,25(OH)_2_D_3_ on gene expression depend on the presence of the VDR. We measured VDR gene expression to find if it was more abundant in the infected mammary gland. VDR expression was slightly higher in mammary tissue from the infected mammary gland than in tissue from the contra-lateral uninfected mammary gland (Fig. 3A). However, VDR expression in the cells isolated from milk of the infected mammary gland was 8 fold higher than VDR expression in cells from the contra-lateral uninfected mammary gland (*P*<0.05) and 75 fold higher than VDR expression in peripheral blood leukocytes (*P*<0.001; Fig. 3B). In the cells from the infected mammary gland, there was no difference of VDR expression between the CD14^+^ and CD14^−^ populations (Fig. 3C). In both populations though, VDR expression was at 50 fold greater in the cells from the infected mammary gland than in peripheral blood leukocytes (*P*<0.05) (Fig. 3C).

**Figure 4:**
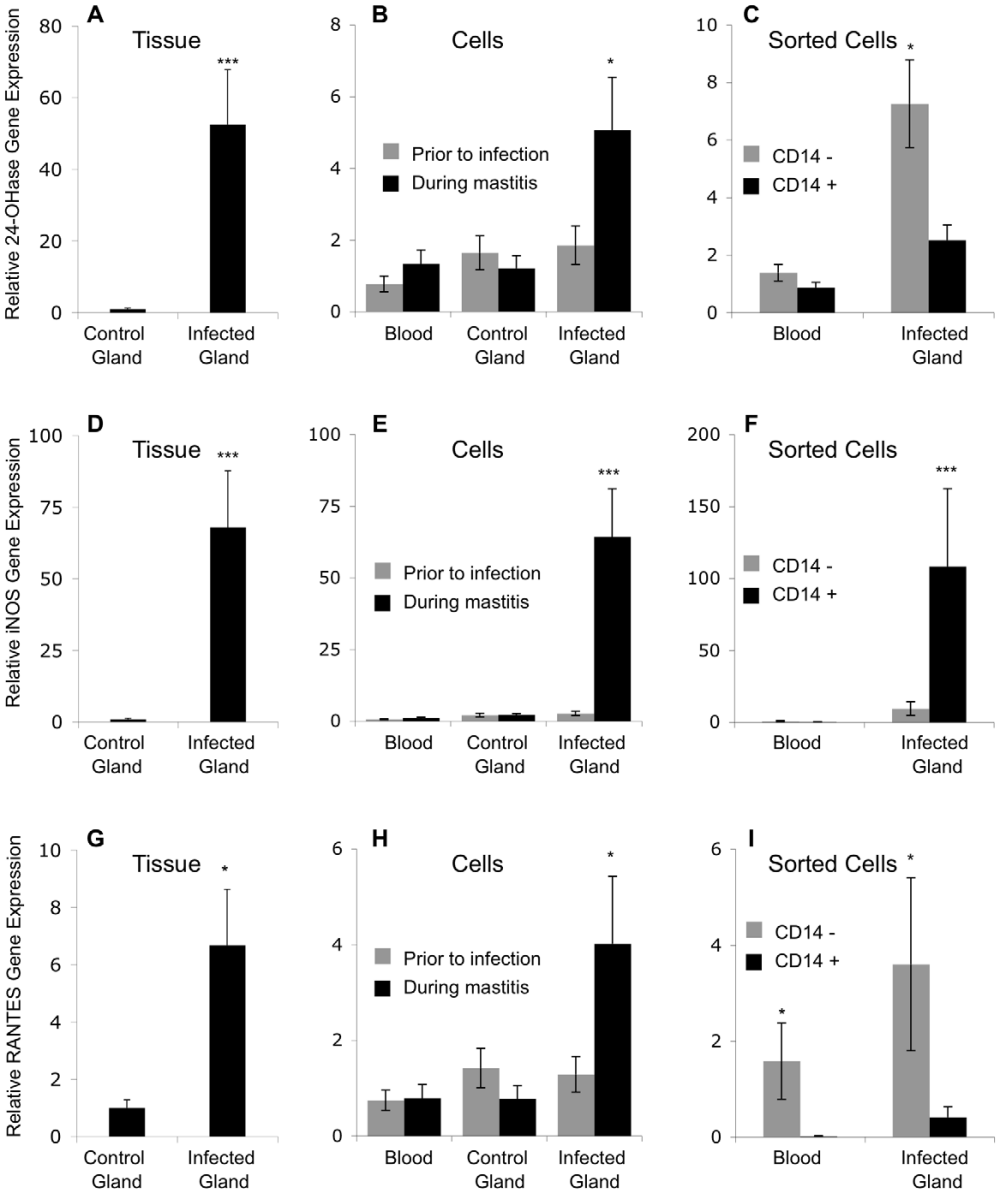
Expresssion of 1,25(OH)_2_D_3_ inducible genes during mammary infection. Relative 24-OHase (**A–C**), iNOS (**D–F**), and RANTES (**G–I**) gene expression in mammary tissue, cells from peripheral blood and cell from milk during mammary infection. Tissue (n = 3 cows) and cells (n = 5 cows) were collected and sorted as in Figure 2. The amount of mRNA for each gene in each sample was determined by quantitative real-time RT-PCR and normalized to RPS9 mRNA. The relative amount of mRNA for each gene was determined using the 2^-ΔΔCt^ method. Data represent the mean ± SE expression of each gene relative to expression in control tissue (**A**), or peripheral blood leukocytes (**B and C**). ANOVA was performed by SAS using the general linear model and multiple comparison tests were made using the Tukey adjustment; mean is different from other means* *P*<0.05, *** P<0.001.

The 24-OHase gene expression was 50 fold higher in the infected mammary glands relative to 24-OHase expression in the contra-lateral uninfected mammary glands (*P*<0.001; Fig. 4A). The 24-OHase expression in cells isolated from milk was 4 fold higher in cells from the infected mammary gland than in cells from the contra-lateral uninfected mammary gland or peripheral blood (*P*<0.05; Fig. 4B). During bacterial infection, 24-OHase expression was not significantly higher in CD14^+^ cells from the infected glands compared to the CD14^+^ cells from blood, but was higher in CD14^−^ cells from the infected glands compared to CD14^−^ cells from blood (*P*<0.05; Fig. 4C).

In vitro activated bovine monocytes, 1,25(OH)_2_D_3_ increases expression of iNOS and RANTES [8]; so, we measured the expression of both genes in mammary tissue and cells from the control and in vivo infected mammary glands (Fig. 4D–I). iNOS and RANTES expression was greater (*P*<0.001 for iNOS and *P*<0.05 for RANTES) in tissue and cells from the infected mammary glands than in tissue or cells from the contra-lateral uninfected mammary glands. Like 1α-OHase, iNOS was predominantly expressed in the CD14^+^ cells from the infected gland (*P*<0.001 compared to CD14^−^ cells from the infected glands). RANTES, however, was expressed more in the CD14^−^ population from the infected glands compared to CD14^+^ cells from the infected glands (*P*<0.05).

## Discussion

Numerous in vitro studies have shown that 1α-OHase expression and subsequent 1,25(OH)_2_D_3_ induction of 24-OHase in monocytes and macrophages is induced by TLR signaling in vitro [6], [8], [19]. However, there was a lack of in vivo evidence that the vitamin D pathway was induced in macrophages in response to infection. Genes of the vitamin D pathway were elevated in macrophages in lesions of leprosy patients [10] but that evidence could not confirm whether or not the pathway was upregulated in response to infection. In this study, we give in vivo confirmation that genes of the vitamin D signaling pathway were upregulated in response to bacterial infection. Furthermore, macrophages at the site of the bacterial infection were the predominant cells that expressed 1α-OHase, which confirms the many in vitro studies that have shown that 1α-OHase is expressed in macrophages upon pathogen recognition.

Induction of 1α-OHase gene expression in the infected mammary gland has major implications because it allows for local control of 1,25(OH)_2_D_3_ synthesis. In addition to upregulation of 1α-OHase, VDR gene expression was elevated in cells from the infected mammary gland. Increased VDR expression would have enhanced the sensitivity of those cells to 1,25(OH)_2_D_3_. Induction of 1α-OHase and VDR expression, consequently allowed for control of vitamin D responsive genes in the infected mammary glands. During bacterial infection, 24-OHase, iNOS and RANTES were expressed in the infected mammary glands. Cathelicidin genes have previously been shown to not be regulated by 1,25(OH)_2_D_3_ in cattle, in contrast to other species, and therefore were not tested [8]. However, induction of 1α-OHase gene expression in bovine monocytes in the presence of 25(OH)D_3_ resulted in upregulation of iNOS and RANTES [8]. Accordingly, upregulation of iNOS and RANTES expression in monocytes is in part depended on local 1α-OHase activity and 1,25(OH)_2_D_3_ production. Likewise, the expression of 24-OHase is known to be highly upregulated by 1,25(OH)_2_D_3_ [23], [24]. The upregulation of 24-OHase in the infected mammary glands indicates that 1,25(OH)_2_D_3_ was synthesized locally in the infected mammary glands. Altogether, our data provides in vivo evidence that a vitamin D signaling mechanism is activated as part of the innate immune response to pathogens in order to provide local control of vitamin D-dependent immune responses.

The substrate for 1α-OHase is 25(OH)D_3_, so production of 1,25(OH)_2_D_3_ and subsequently regulation of gene expression by 1,25(OH)_2_D_3_, depends on the availability of 25(OH)D_3_. The circulating concentration of 25(OH)D_3_ depends on dietary intake of vitamin D and exposure to sunlight [25], [26]. In cattle supplemented with the recommended amount of vitamin D, the circulating concentration of 25(OH)D_3_ typically ranges from 20 to 50 ng/mL [27]. Circulating concentrations of 25(OH)D above 20 ng/mL have been considered adequate for calcium homeostasis [28], however, circulating 25(OH)D concentrations below 30 ng/mL are now considered insufficient for proper immune function in humans [1], [29], [30]. The target range for 25(OH)D in humans still remains elusive, however, because of the inability to perform tightly controlled experiments with human subjects. Similarities between cattle and humans in regards to vitamin D metabolism and immune function [31], [32], [33] indicate that cattle are a useful model to study vitamin D requirements for proper immune function in humans. Experiments to find an optimal range of circulating 25(OH)D for proper immune function in cattle, however, have not been performed. Therefore, efforts to find the optimal range of 25(OH)D concentration for proper immune function in cattle has implications for bovine and human health.

We did measure the concentration of 1,25(OH)_2_D in milk during mammary infection but the there was not a detectable increase of 1,25(OH)_2_D in milk from the infected mammary gland compared to milk from an uninfected mammary gland. In fact milk 1,25(OH)_2_D_3_ was at or below the detection limits of the assay (data not shown) This was expected as the concentration of vitamin D metabolites is are low in milk compared to plasma [27], [34], and the 1,25(OH)_2_D_3_ produced by macrophages would have been diluted as milk accumulated in the cistern of the mammary gland.

The mean blood concentration of 25(OH)D in the cows used in this study was 74.5 ng/ml (SEM +/−4.5), which is above the 25OHD_3_ threshold needed for innate immune cells to produce 1,25(OH)_2_D_3_ [8], [35]. The increased expression of the 1α-OHase we observed in innate immune cells from infected mammary tissue along with the subsequent induction of 24-OHase in these cells supports the conclusion of local in vivo production of 1,25(OH)_2_D_3_ during an infection. We show for the first time that an in vivo infection results in activation of vitamin D with the concomitant downstream vitamin D dependant genes of the innate immune system activated similar to that previously observed in vivo.
